# Unravelling Diurnal Asymmetry of Surface Temperature in Different Climate Zones

**DOI:** 10.1038/s41598-017-07627-5

**Published:** 2017-08-04

**Authors:** R. Vinnarasi, C. T. Dhanya, Aniket Chakravorty, Amir AghaKouchak

**Affiliations:** 1 Department of Civil Engineering, Indian Institute of Technology Delhi, Hauz Khas, New Delhi India; 2 Centre for Hydrology & Remote Sensing (CHRS), Department of Civil & Environmental Engineering, University of California, Irvine, California USA

## Abstract

Understanding the evolution of Diurnal Temperature Range (DTR), which has contradicting global and regional trends, is crucial because it influences environmental and human health. Here, we analyse the regional evolution of DTR trend over different climatic zones in India using a non-stationary approach known as the Multidimensional Ensemble Empirical Mode Decomposition (MEEMD) method, to explore the generalized influence of regional climate on DTR, if any. We report a 0.36 °C increase in overall mean of DTR till 1980, however, the rate has declined since then. Further, arid deserts and warm-temperate grasslands exhibit negative DTR trends, while the west coast and sub-tropical forest in the north-east show positive trends. This transition predominantly begins with a 0.5 °C increase from the west coast and spreads with an increase of 0.25 °C per decade. These changes are more pronounced during winter and post-monsoon, especially in the arid desert and warm-temperate grasslands, the DTR decreased up to 2 °C, where the rate of increase in minimum temperature is higher than the maximum temperature. We conclude that both maximum and minimum temperature increase in response to the global climate change, however, their rates of increase are highly local and depend on the underlying climatic zone.

## Introduction

Global warming due to the rise in greenhouse gas emissions is usually quantified through the long-term changes in the mean temperature (*T*_*mean*_) of the earth’s surface, expressed in yearly, decadal or even centurial timescales. However, regional scale impact assessment, adaptation, and mitigation of climate change need an evaluation of suitable climate change indicators at daily time scales. Diurnal Temperature Range (DTR), defined as the difference between daily maximum and minimum temperatures, is a key driving factor in most of the climatological processes^1–3^ and is an important index of diurnal variations^4^. The intensity, frequency, and duration of extreme precipitation events are increasing, on average, with a decrease in DTR due to increase in night time temperatures than daytime temperatures^5–9^. Further, any change in DTR would increase the risks associated with drought^10^, heat stress^11^, crop failure^12, 13^, human health^14^ and mortality rate^15^.

Numerous studies^1, 16, 17^ reported a global decrease in DTR at a rate of around 0.07 °C per decade^18^, with the decrease being evident during 1950–1980^19, 20^ and attributed this to the higher rate of increase of minimum temperature (*T*_*min*_) than maximum temperature (*T*_*max*_). However, a few regional studies, mainly in the tropics and subtropics, reported an increasing trend^21, 22^ in DTR and attributed it solely to the increase in *T*_*max*_, which may be ascribed to the influence of regional climate on the behaviour of DTR. Further, ref. 20 highlighted that the acuteness of DTR is uncertain, especially in the south Asian regions such as India and China, arising mainly due to the unavailability of reliable regional data products. In this study, we analysed the evolution of *T*_*mean*_, *T*_*max*_, *T*_*min*_ and DTR over India using the recently available 1°×1° gridded daily temperature data provided by Indian Metrological Department (IMD)^23^. Detailed analysis of DTR is crucial over this region owing to the associated risks^12, 13, 15^, aggravated by an agriculture-dependent economy and dense population. Spatio-temporal exploration of DTR in different heterogeneous climatic zones^24^ in India, i.e., arid/semi-arid in north-west, warm-temperate in central India, sub-tropical humid in north-east and foothills of Himalayas, semi-arid and equatorial grassland in southern India and warm-humid coastal regions, is expected to reveal the generalized influence of regional climate on DTR.

Past studies over India^2, 17, 25^ attributed an increasing trend in DTR to an increase in *T*_*max*_ and no/insignificant change in *T*_*min*_. On the contrary, few studies^26, 27^ observed a decreasing trend in both *T*_*min*_ and *T*_*max*_ over western Himalayas. Further, ref. 28 used high-resolution temperature data and observed no significant trend in DTR, except significant decrease over northernmost India. These contradictions can be attributed to the limitations in traditional trend analysis. Moreover, traditional statistical trend approaches employed in the past consider either a constant linear trend or a shape established *a priori* ^29^. Such approaches are sensitive to the boundaries of the time series and hence fail to capture the multi-decadal variability^30^. To eliminate these disadvantages and to understand the non-stationary behaviour, we use Multidimensional Ensemble Empirical Mode Decomposition (MEEMD)^31^, which is an improved method for scientific data analysis (See Methods). Considering the availability of data and in order to compare the present study with the previous studies^27, 32^, we restrict the analysis to a period of 60 years from 1951 to 2010.

## Result and Discussion

**Figure 1:**
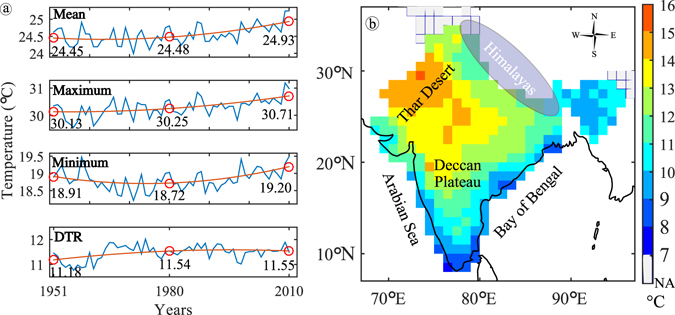
(**a**) Annual variation of spatially averaged annual temperature over India and (b) Average DTR from 1951 to 2010. Grids with inconsistent data are shown in grey colour (extreme bottom of colour bar), which is represented as NA (Not Analysed). The maps were generated using the software MATLAB (version 2014b). http://www.mathworks.com/products/matlab/.

**Figure 2:**
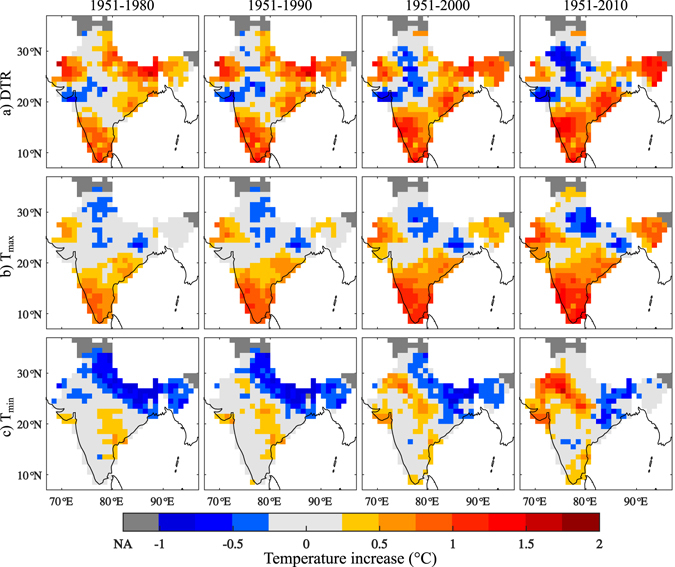
Spatial evolution of Ensemble Empirical Mode Decomposition trend of annual (**a**) DTR, (**b**) Maximum and (**c**) Minimum temperatures. Each sub panel has four time windows 1) 1951–1980, 2) 1951–1990, 3) 1951–2000 and 4) 1951–2010. Grids with inconsistent data are shown in dark grey colour (extreme left of colour bar), which is represented as NA (Not Analysed). ±0.25 °C range is assigned a light grey colour for easy distinction of positive and negative trends. The maps were generated using the software MATLAB (version 2014b) http://www.mathworks.com/products/matlab/.

The regional average of *T*_*mean*_, *T*_*max*_, *T*_*min*_ and DTR over India is shown in Fig. 1a. *T*_*mean*_ over India shows an increasing trend since 1951. An identical pattern is observed for *T*_*max*_. However, *T*_*min*_ decreases slightly till 1980 with a magnitude of 0.19 °C, which results in subsequent increase (0.36 °C) in DTR. Thereafter, insignificant trend in DTR can be attributed to the rapid increase in *T*_*min*_. Figure 1b shows the 60-year average of DTR, which ranges from 7 °C to 16 °C, with its values gently increasing from humid coastal regions to north-western arid regions. To understand the temporal evolution of temperature, we evaluated the spatial EEMD-trend (See Methods for Definition) of annual DTR, *T*_*max*_, and *T*_*min*_ for four distinct time windows (1951–1980, 1951–1990, 1951–2000 and 1951–2010), as shown in Fig. 2. DTR (Fig. 2a, top panel) shows a positive trend over southern west coast, foothills of the Himalayas and some parts of north-west, which is analogous to results from a previous study^32^. The spatial extent of positive trend is found to gradually increase over the Equatorial and north-eastern regions, with an approximate increase of 0.25 °C per decade. On the contrary, significant negative trend is observed over some parts of northern west coast during the initial 30 years, which has gradually extended towards central and northern India during the subsequent decades. The spatial extent and magnitude of the positive trend over the north-west also decreases over the decades. Negative trends in DTR over north-west and central India, known for its arid/semi-arid climate, well agree with the behaviour of climatologically similar regions taken up by previous studies^33, 34^. A similar analysis of *T*_*max*_ reveals the substantial increase in the spatial extent and the magnitude of the positive trend over the southern west coast (Fig. 2b, middle panel). *T*_*max*_ of the entire coastal region is increasing at a rate of 0.25 °C per decade. Similar intensification of warming is visible in the north-east during the recent decades. This explains the increase in DTR over these regions. However, a reversal of trend pattern is observed for *T*_*min*_ (Fig. 2c, bottom-panel). While, the negative trend of *T*_*min*_ is manifested in the foot-hills of Himalayas^26^ in the initial years, it has shrunk gradually over the decades, along with the concurrent evolution of its positive trend in the north-west after 1990. Interestingly, positive and negative trend patterns of DTR are consistent with the positive trends in *T*_*max*_ and *T*_*min*_, respectively^27^.

**Figure 3:**
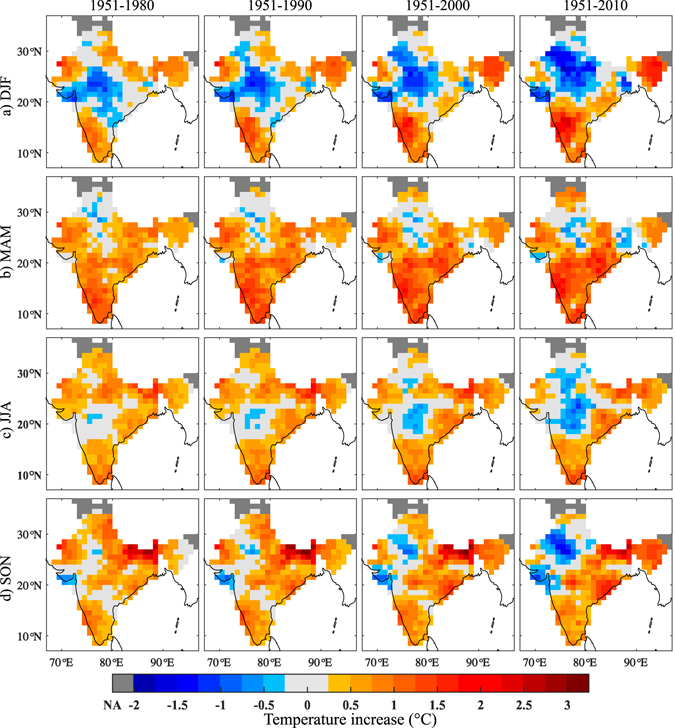
Spatial evolution of Ensemble Empirical Mode Decomposition trend of seasonal DTR for (**a**) DJF, (**b**) MAM, (**c**) JJA and (**d**) SON. Each sub panel has four time windows 1) 1951–1980, 2) 1951–1990, 3) 1951–2000 and 4) 1951–2010. Grids with inconsistent data are shown in dark grey colour (extreme left of colour bar), which is represented as NA (Not Analysed). ±0.25 °C range is assigned a light grey colour for easy distinction of positive and negative trends. The maps were generated using the software MATLAB (version 2014b) http://www.mathworks.com/products/matlab/.

We also explored the DTR trends in different seasons to understand the influence of seasons on DTR in various regions. MEEMD analysis is repeated for four prominent seasons, i.e., winter (DJF: December to February), pre-monsoon (MAM: March to May), summer monsoon (JJA: June to August) and post-monsoon (SON: September to November) as shown in Fig. 3. During winter, DTR shows negative trend in central India and few parts of the north-west; and positive trend in west coast and the north-east^32^ (Fig. 3a, top panel). The spatial extent of this negative trend has moved towards the entire north-west and central India over the decades, with a decrease of at most 2 °C in most of these regions by 2010. Likewise, the positive trend of DTR in west coast has increased by at most 3 °C covering the entire Peninsular India. Pre-monsoon season shows a constant positive change in DTR in most of the grids (Fig. 3, 2^nd^ panel). It is evident that there is no significant change, except approximately 0.5 °C/decade increase in a few grids of Deccan Plateau. Positive trend dominates during monsoon season too, except over central India. However, a manifestation of considerable evolution is absent over southern India, east coast and north-east (Fig. 3, 3^rd^ panel). Moreover, a reversal of trend is seen over the entire northern India by 2010. The trend in the post-monsoon period (Fig. 3, bottom panel), follows the same pattern as that of the winter with more dominance of positive trend over the region.

**Figure 4:**
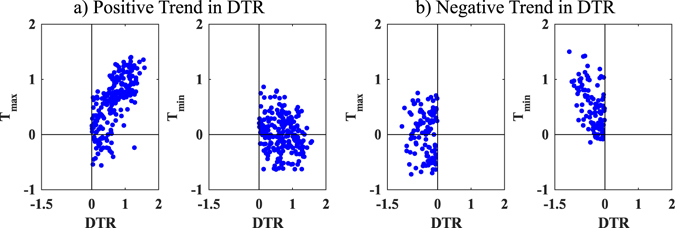
Scatter plot of Ensemble Empirical Mode Decomposition trend in a) positive trend of DTR and *T*_*max*_ (*T*_*min*_) and b) negative trend of DTR and *T*_*max*_ (*T*_*min*_) for the last time window (1951–2010). Unit is in °C. The maps were generated using the software MATLAB (version 2014b) http://www.mathworks.com/products/matlab/.

The seasonal variations in DTR are further understood with an investigation of the corresponding variation of *T*_*max*_ and *T*_*min*_ (Figures S2 and S3). This reveals that the EEMD-trend is similar to that of annual pattern, with different spatial extent and magnitude (for a detailed description see Supplementary Section-2). Especially, the increase in *T*_*min*_ (~3 °C after 1950, Figure S3) during winter, particularly in the agriculturally rich areas of north-west, will severely affect the yield of winter crops^12^ and the warming phase of *T*_*min*_, may also affect the yield of paddy in other parts of India, if prolonged^13^. Intensification of warming, irrespective of seasons, is apparent from the spatial pattern of various aspects of *T*_*max*_ and *T*_*min*_ extremes, computed using extreme event indices defined by CLIMDEX (see Table S1). High (low) extremes of *T*_*max*_ and *T*_*min*_ are found to be following an increasing (decreasing) trend over the decades (Figures S4 and S5). This suggests that both *T*_*max*_ and *T*_*min*_ are increasing, however, their respective rate of increase determines the DTR trend. This relationship between DTR and the temperature extremes is further analysed through scatter plots of DTR against the respective *T*_*max*_ and *T*_*min*_ for the grids with positive and negative trends separately (Fig. 4). It shows that the positive trend in DTR is because of a higher rate of increase in *T*_*max*_ than *T*_*min*_, while the reverse is also true for the negative trend of DTR.

**Figure 5:**
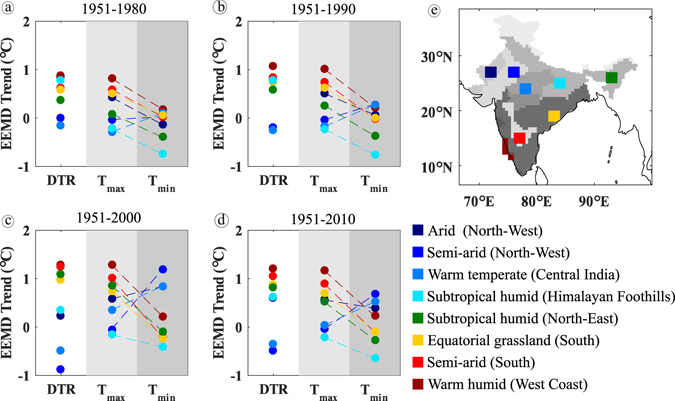
Regional Ensemble Empirical Mode Decomposition trend of DTR, *T*_*max*_ and *T*_*min*_ for heterogeneous climatic zones (**a**) 1951–1980, (**b**) 1951–1990, (**c**) 1951–2000 and (**d**) 1951–2010, (**e**) representative grids in different climatic zones. Different grey colours in India map shown in panel ‘e’ are indicative of different climatological regions of the representative grids. The maps were generated using the software MATLAB (version 2014b) http://www.mathworks.com/products/matlab/.

The influence of regional climate on the relationship between DTR, *T*_*max*_, and *T*_*min*_ is investigated by comparing their EEMD-trends for different climatic zones and for different time windows (Fig. 5). For this analysis, representative grids were taken from these climatic zones. We observed that the north-western arid and semi-arid regions; and foothills of Himalayas showed a strengthening of negative DTR trends with an increase in the time window, which is because of a strengthening of positive trends in *T*_*min*_ compared to *T*_*max*_, likewise the reverse is also true. Regions with tendency of DTR towards positive trends (north-east, semi-arid Deccan plateau, Equatorial grasslands and west coast) owe it to the tendency of *T*_*max*_ to move towards positive trends compared to *T*_*min*_. The increase in DTR caused by the increase in *T*_*max*_ can result in precipitation deficit^6, 10^. Especially in north-east and the Western Ghats, which are high rainfall receiving regions, the positive trend of DTR may signal the increase in dry days^30^. This suggests that the difference in the regional evolution of DTR is influenced by the different regional response of *T*_*max*_ and *T*_*min*_. Clearly, *T*_*min*_ is increasing at a faster pace in the north-west. Even the magnitude of the negative trend at other places has significantly reduced over the decades. Traditional methods of linear trend analysis fail to capture this increasing phase^2, 26, 27^. For statistical justification, the significance of trends are checked at 10% significance level and the grids showing significant trends for all variables are shown in Figure S6.

The regional patterns of DTR depend on the rate of increase in *T*_*min*_ and *T*_*max*_. In India, the regional climate affects this rate of increase through many different factors like cloud cover^1, 2, 12, 28^, precipitation^1, 2, 34^, soil moisture^1, 2^, vegetation cover^34^, etc. All the above processes are inversely correlated with DTR. However, these feedback processes are local in nature, which are consequently stimulated by some global factors. For instance, DTR over northern India decreases with increase in cloud cover formed due to aerosol loading^2, 12, 28, 35^. However, a contradictory pattern of DTR is observed over southern India under high cloud cover^12, 28^. This behaviour can be attributed to the proximity to the sea and the rising trend of Sea Surface Temperature (SST)^12^, as significant positive correlation between SST and near surface air temperature exists^35, 36^. Moreover, we observe an increasing trend of near surface air temperature in coastal regions, where the effect of urbanization is not pertinent^32^. To understand this behaviour of diurnal temperature variations, we also analysed the SST (see supplementary information). Since SST directly depends on the ocean heat content, which is affected by the global mean temperature^37^, it is pertinent to suggest that the effect of the global climate on SST is transferred to the observed changes in the land surface temperature. Figure S7 reveals that the rate of warming of the Arabian Sea is higher than the Bay of Bengal, which could explain the evolution of DTR from west to east over time. The difference in the SST of Arabian Sea and Bay of Bengal can impact the surface temperature behaviours over land, while it is also affected by the local factors like vegetation cover of the region^38^. The increase in humidity in the arid deserts and warm-temperate grasslands due to the warming of the Arabian Sea and increase in cropland cover^39^ can explain the significant increase in *T*_*min*_, which leads to a decrease in DTR in these regions. In case of west coast, the warmed up sea causes both *T*_*max*_ and *T*_*min*_ to rise, however the higher rate of increase in *T*_*max*_ than *T*_*min*_ can be attributed to the decline in forest cover. The diurnal asymmetry pattern over the sub-tropical forest in the north-east can be attributed to the warming up of Bay of Bengal. From the above discussion, it is evident that the rise in both *T*_*min*_ and *T*_*max*_ in all climatic zones is in line with global climate change, whereas the rate at which they increase is strongly influenced by the regional factors, with clear distinction among the climate zones. However, a detailed study investigating the factors affecting DTR changes is needed to better understand this behaviour of diurnal temperature variations over India.

## Conclusion

This study presents the analysis of Diurnal Temperature Range (DTR) and temperature employing a non-stationary and non-linear approach known as Multi-dimensional Ensemble Empirical Mode Decomposition (MEEMD) method, using gridded daily temperature data of past 60 years (1951–2010) over India. To further understand the influence of regional climate on DTR, spatio-temporal exploration of DTR over different heterogeneous climatic zones are performed. The following conclusions are derived from this study:

- The regions conventionally showing high DTR such as arid, semi-arid and warm temperate grasslands of India, show negative trend in DTR, because the rate of increase in minimum temperature is higher than that of the maximum temperature.
- Sub-tropical forests, equatorial grasslands down south and the west coast show a higher rate of increase in maximum temperature with no/negligible change in minimum temperature, resulting in positive trend of DTR.
- The season-wise analysis reveals that the evolution in trend of DTR is predominantly evident during winter (DJF) and post-monsoon (SON) seasons, indicating warmer winters.
- Analysis of extremes shows an increasing (decreasing) warm (cold) days and nights. In addition, the regional trend of minimum temperature suggests a shift towards global pattern of increasing night time temperature, contrasting the pattern reported in the recent literature.
- The increase in minimum, maximum and mean temperatures can be attributed to global climate change. However, the changes in DTR mainly depend on the relative rate of increase in maximum and minimum temperatures, which is chiefly dominated by the regional climate.

While previous studies mainly attributed DTR changes to local factors^1, 2, 28^, our analysis on DTR over India with SST reveals that both global and local factors are working in tandem. We argue that the significant decrease in DTR in arid desert and warm-temperate grasslands can be attributed to the global influence on the Arabian Sea and subsequent increase in humidity over these regions. However, the rate of increase of *T*_*min*_, which is a factor of local micro-climate, also plays a dominating role leading to a negative trend in DTR over these regions. In case of the west coast, the Arabian Sea warming has a direct influence on the observed positive trend in DTR since the warmed up sea causes the *T*_*max*_ to rise faster than the *T*_*min*_. However, plausible the above reasons may sound, further detail analysis is required to obtain a clear picture of the factors influencing diurnal asymmetry, both local and global.

## Methods

### Data Description

High resolution, 1° × 1° gridded, daily maximum, minimum and mean temperature prepared by National Climatic Centre (NCC), Indian Meteorological Department (IMD) is used in this study^23^. The spatial domain of this gridded data set is 7.5°N to 37.5°N and 67.5°E to 97.5°E, covering the main land region of India. After proper quality checks, the daily temperature record obtained from 395 stations are selected for the period of 1951–2013. Further, the most appropriate interpolation technique for irregular spaced data^40^, which is, modified version of Shepard’s angular distance weighing algorithm^41^, is used to convert the station data into gridded data. The accuracy of the gridded dataset evaluated using cross-validation and the RMSE (Root Mean Square Error) was found to be lesser than 0.5 °C. Moreover, the present dataset is compared with the monthly mean temperature dataset prepared University of Delaware^42^ and correlation coefficient is noted to be around 0.8 in most of the grids^23^. This dataset is found to be useful for extreme temperature analysis, since the occurrence of historical heat waves exactly associate with the present data set. The all-India average of maximum, minimum and mean temperature varies from 20.48 °C to 34.18 °C, 7.7 °C to 24.4 °C and 14.1 °C to 28.9 °C respectively. This data set is extensively used in the literature, especially for analysis of extremes^11, 43^.

### Extreme Event Indices

The extreme event indices adopted in the analysis have been defined based on the ‘Expert Team on Climate Change Detection and Indices’ (ETCCDI) recommendation^44^ (available from http://www.climdex.org/indices.html). Totally 9 indices are analyzed out of 16 indices proposed^44^ by ETCCDI. The other indices such as GSL (Growing Season Length), FD (Number of Frost Days), ID (Number of Icing Days) are not suitable indices for Indian weather. Instead of taking the extremes of maximum and minimum temperature, we calculated the average annual/seasonal maximum and minimum temperature to analyze the changes in DTR. The percentile threshold value is defined as the calendar day 90^th^ percentile centered on a 5-day window for the base period 1961-1990. Bootstrap approach is used to calculate the number of days in the base period to avoid homogeneity in the in-base and out-base period^45^. Indices used in this study are shown in Table S1.

### Multidimensional Ensemble Empirical Mode Decomposition

Multidimensional Ensemble Empirical Mode Decomposition (MEEMD)^31^ is a method for eliminating the oscillatory component of a time series and revealing while preserving the slow varying component^46^. This approach does not need a functional form *a priori* and is capable of extracting the hidden nonlinear and non-stationary nature of the time series^29, 47^. In MEEMD, a time series, *Y*(*t*), at a grid point, is decomposed using EEMD^46, 48^ into oscillatory components known as Intrinsic Mode Functions (IMF) as shown in equation (1). The sequential elimination of IMFs (*I*_*j*_) produces residual (*R*_*n*_), which is either monotonic or contains only one extremum, and cannot be further decomposed into an oscillatory component.

1 $$Y(t)=\sum _{j=1}^{n}{I}_{j}(t)+{R}_{n}(t)$$

A detailed methodology is presented in the Supplementary Section-1. MEEMD is less sensitive to the boundary compared to the other commonly used methods, hence the physical meaning of the time series will be retained even after appendage of data^49^. MEEMD has been successfully implemented in various climate studies^29, 49^ due to its effectiveness and the spatial-temporal locality. Since the trend varies with time, as described above, the averaged slope of the trend within a time window cannot appropriately capture the evolution of the trend. Hence, the trend at a particular time(*t*) is represented as the increment of the EEMD-trend (Residual)^29^ from that of the reference time (1951) as shown in equation (2).

2 $${{\rm{Trend}}}_{{\rm{EEMD}}}(t)={R}_{n}(t)-{R}_{n}(1951)$$

### Test for Significance

The statistical significance of these EEMD trends at a given spatio-temporal locations is evaluated by Monte Carlo Simulation adopted by ref. 29. Here, 10000 samples of red noise series were generated, having the same temporal length (60) and lag-1 auto-correlation as that of the original series. The slow varying component of each of the generated series is computed using MEEMD approach and the probability density function (PDF) of the EEMD. The trend at any time is calculated. Then the trend value (ratio between EEMD and standard deviation of the respective variable) of the particular grid is checked whether it is within 10% significance level of the empirical PDF. If it is satisfied, then the EEMD trend is considered to be statistically significant. However, statistically non-significant trend does not mean a complete rejection of the null hypothesis, but only indicates that there is no clear evidence for a trend.

### Data Availability statement

The data that support the findings of this study are available from Indian Meteorological Department (IMD). More details are available in http://www.imd.gov.in/advertisements/20170320_advt_34.pdf.

## Electronic supplementary material

Supplementary Information
