# Supplementary material for: Unravelling Diurnal Asymmetry of Surface Temperature in Different Climate Zones
Source: Sci Rep. 2017 Aug 4;7:7350. doi: 10.1038/s41598-017-07627-5 (PMC5544675; doi:10.1038/s41598-017-07627-5)
Supplement: Supplementary file 1 — Supplementary Information [file 41598_2017_7627_MOESM1_ESM.pdf]

Unravelling Diurnal Asymmetry of Surface Temperature in Different Climate Zones

Supplementary Information

R. Vinnarasi[1], C.T. Dhanya[1], Aniket Chakravorty[1], Amir AghaKouchak[2]

[1]Department of Civil Engineering, Indian Institute of Technology Delhi, Hauz Khas, New Delhi, India.

[2]Centre for Hydrology & Remote Sensing (CHRS), Department of Civil & Environmental Engineering, University of California, Irvine.

Contact information:

Email: dhanya@civil.iitd.ac.in

Office Tel.: +91 11 2659 7328

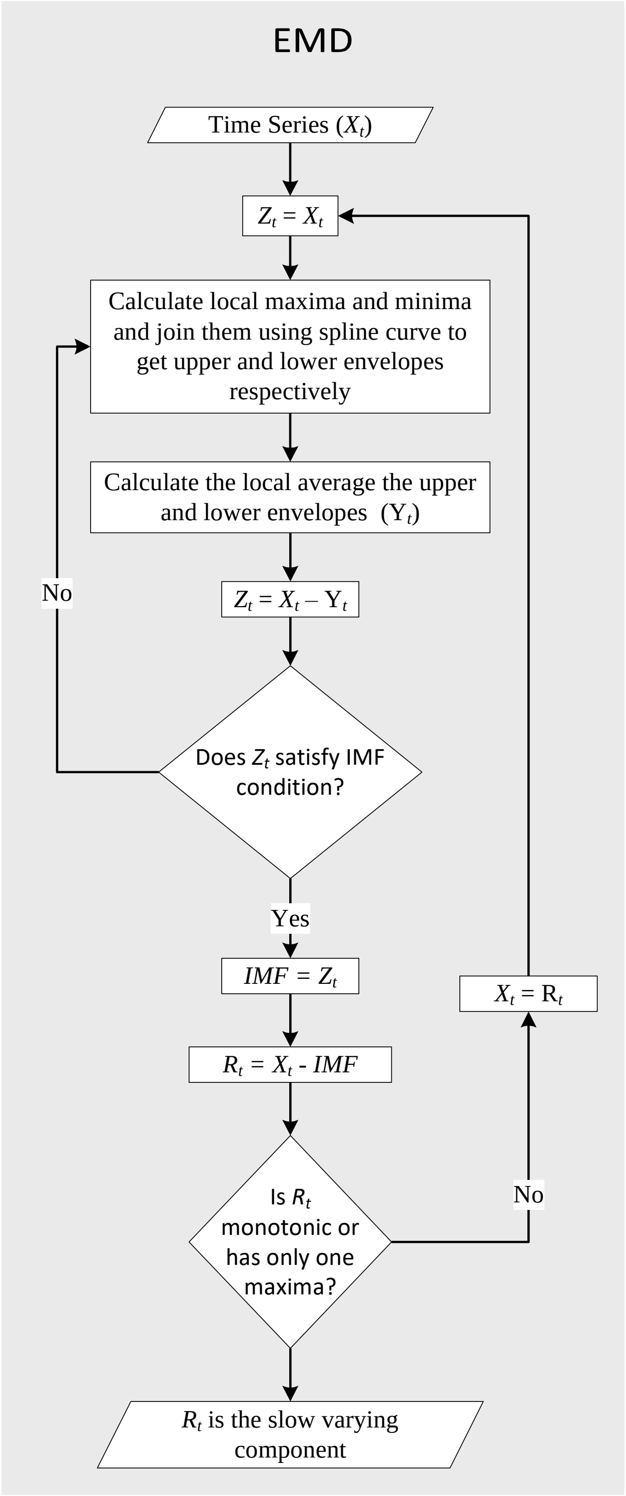

Figure S1: Steps involved in Empirical Model Decomposition of a time series

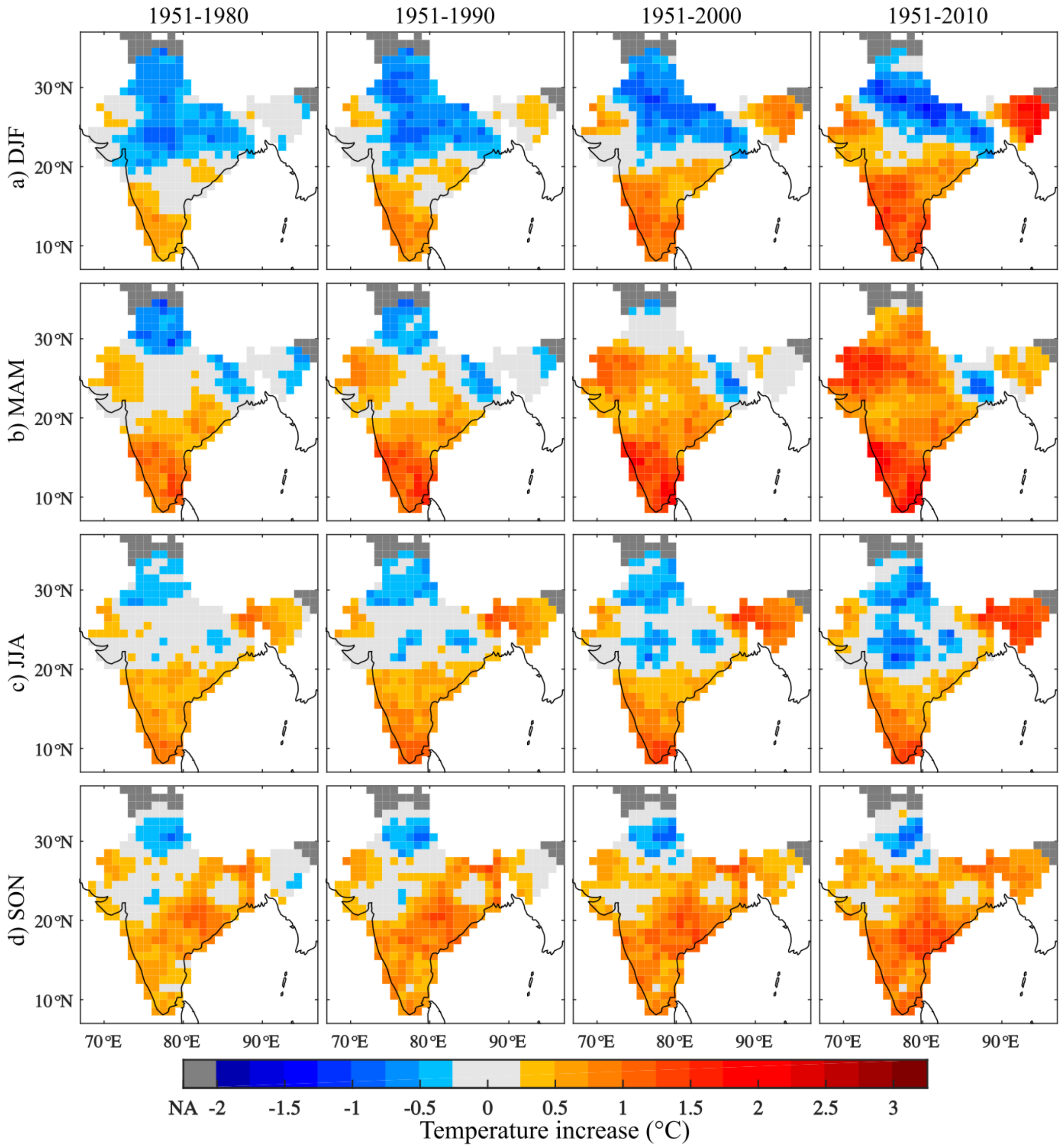

Figure S2: Spatial evolution of Ensemble Empirical Mode Decomposition trend of seasonal maximum temperature for a) DJF, b) MAM, c) JJA and d) SON. Each sub panel has four windows 1) 1951-1980, 2) 1951-1990, 3) 1951-2000 and 4) 1951-2010. Grids with inconsistent data are shown in dark grey color (extreme left of color bar), which is represented as NA (Not Analyzed). ±0.25 ˚C range is assigned a light grey color for easy distinction of positive and negative trends. The maps were generated using the software MATLAB (version 2014b) <http://www.mathworks.com/products/matlab/>

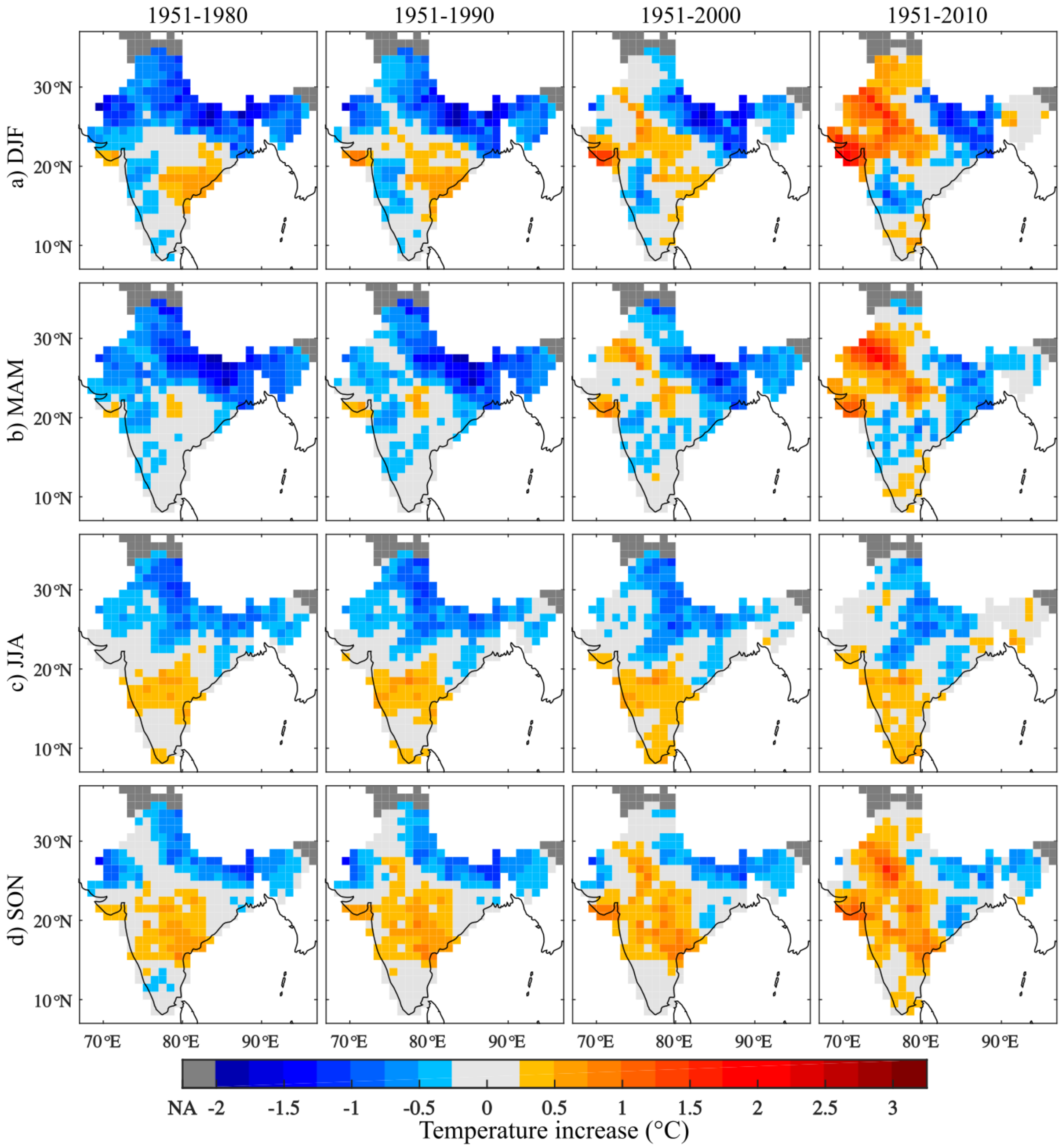

Figure S3: Spatial evolution of Ensemble Empirical Mode Decomposition trend of seasonal minimum temperature DTR for a) DJF, b) MAM, c) JJA and d) SON. Each sub panel has four windows 1) 1951-1980, 2) 1951-1990, 3) 1951-2000 and 4) 1951-2010. Grids with inconsistent data are shown in dark grey color (extreme left of color bar), which is represented as NA (Not Analyzed).± ±0.25 ˚C range is assigned a light grey color for easy distinction of positive and negative trends. The maps were generated using the software MATLAB (version 2014b) <http://www.mathworks.com/products/matlab/>

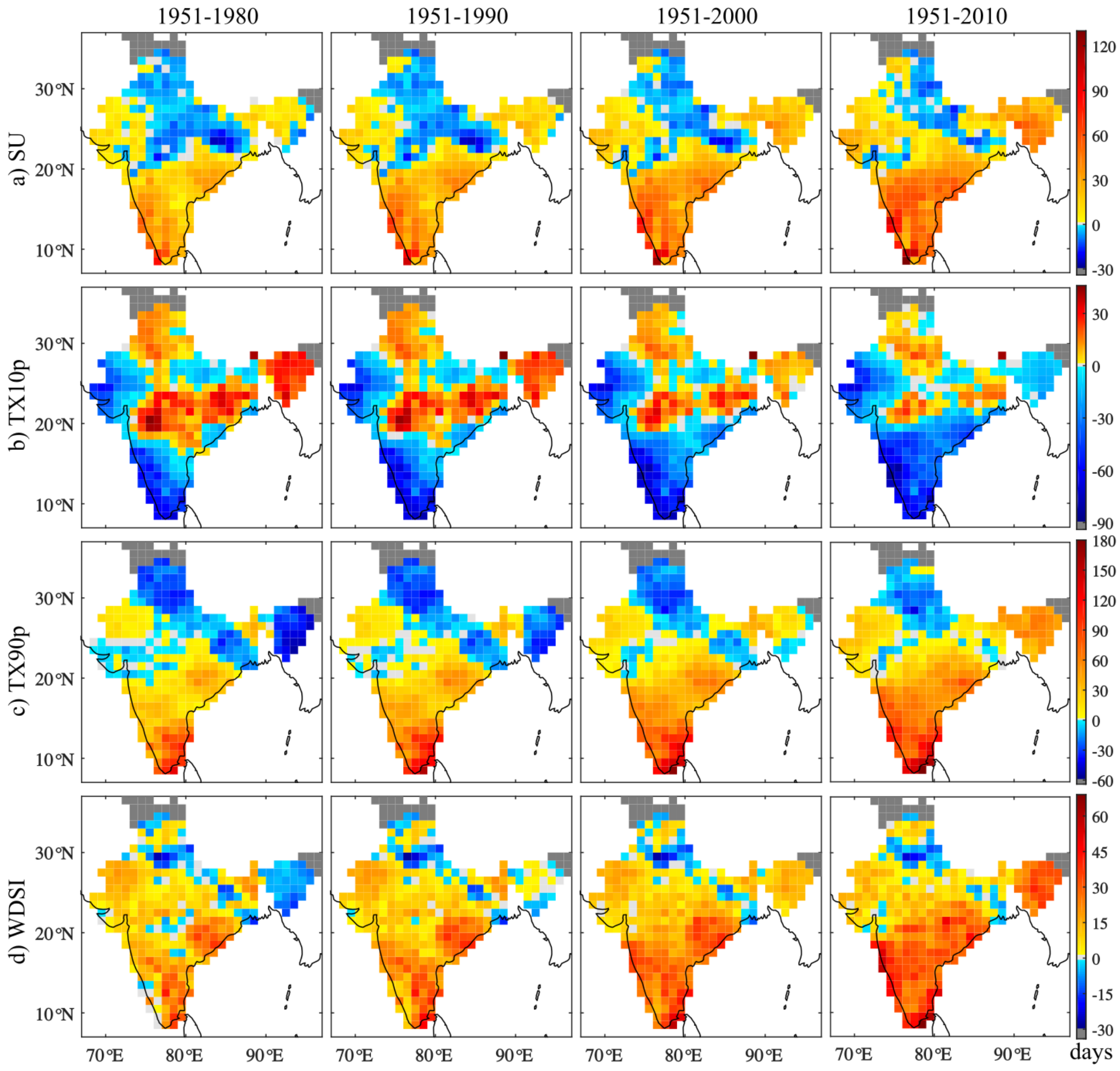

Figure S4: Ensemble Empirical Mode Decomposition trend of a) SU (days), b) TX10p (days) c) TX90p (days) and d) WSDI (days). Each sub-panel has four windows 1)1951-1980, 2)1951-1990, 3)1951-2000 and 4)1951-2010. Grids with inconsistent data are shown in dark grey color in below of the each color bar. ± 1 day range is assigned a light grey color for easy distinction of positive and negative trends. The maps were generated using the software MATLAB (version 2014b) <http://www.mathworks.com/products/matlab/>

Annual occurrence of SU shows positive trend in south India and north-east while negative trend is seen in north India. However, over the decades the spread of negative trend has

decreased and positive trend has evolved with increase in magnitude and extent. Likewise, the trend of cold days and warm days show large spatial extent of negative and positive trend respectively. Though some grids in northern India shows reverse pattern, the evolution of these trends clearly reveals the decrease (increase) in the annual occurrence of cold days (warm days).

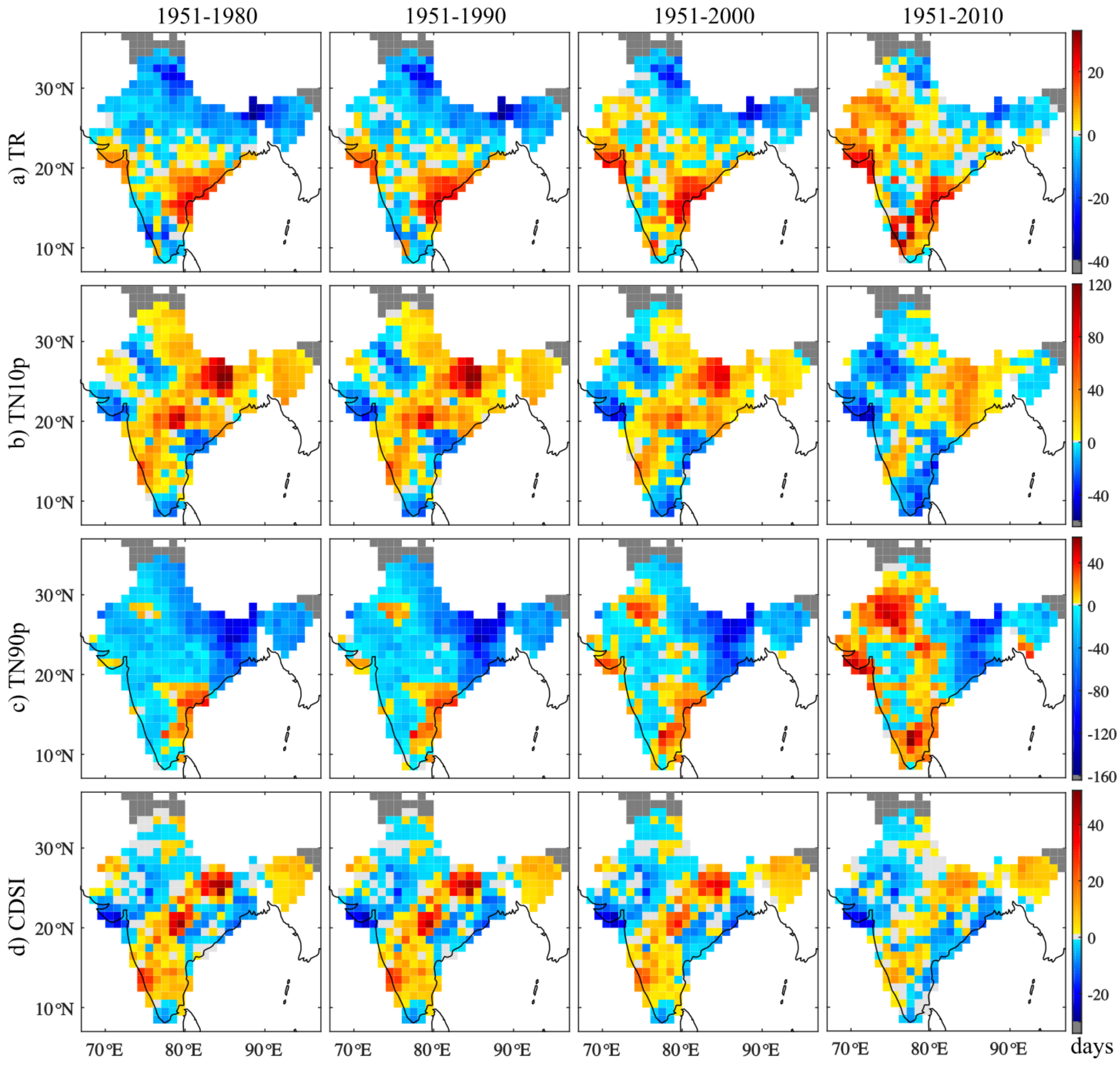

Figure S5: Ensemble Empirical Mode Decomposition trend for the annual occurrence of a) TR (days), b) TN10p (days) c) TN90p (days) and d) CSDI (days). Each sub-panel has four windows 1)1951-1980, 2)1951-1990, 3)1951-2000 and 4)1951-2010. Grids with inconsistent data are shown in dark grey color in below of the each color bar. ± 1 day range is assigned a light grey color for easy distinction of positive and negative trends. The maps were generated using the software MATLAB (version 2014b) <http://www.mathworks.com/products/matlab/>

Except for east Coast of India, negative trend of TR has been observed in first window for the other regions. However, over the decades positive trend has evolved in north-west with

increase in magnitude and extent. Likewise, the trend of cold nights and warm nights show increase in spatial extent of negative and positive trends respectively. Though some grids in eastern part of north India show reverse pattern, the evolution of these trends clearly reveals the decrease (increase) in the annual occurrence of cold nights (warm nights).

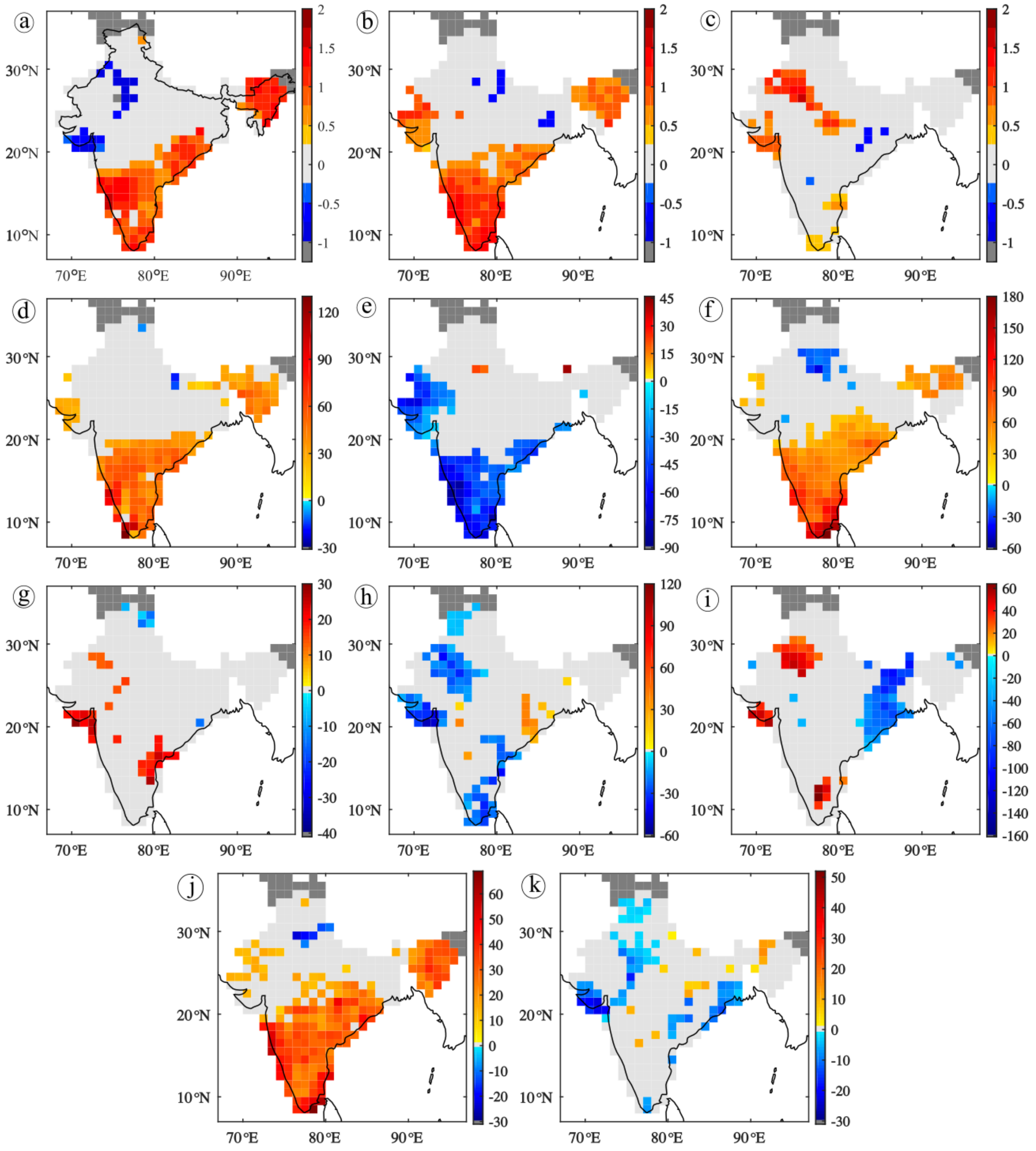

Figure S6: Statistically significant Ensemble Empirical Mode Decomposition trend of a) DTR (°C), b) average maximum temperature (°C) c) average minimum temperature (°C), d) SU (days), e) TX10p (days), f) TX90p (days), g) TR (days), h) TN10p (days), i) TN90p (days), j) WSDI (days) and k) CSDI (days) for 1951-2010 (final time window). Grids with inconsistent data are shown in dark grey color in below of the each color bar. Statistical significance is estimated for 10% significance level using two tailed hypothesis test.

northeastThe maps were generated using the software MATLAB (version 2014b)

<http://www.mathworks.com/products/matlab/>

The statistical significance of the trend is evaluated, for the last window (1951 to 2010), for all the variables, at 10% significance level, using two tailed hypothesis test. The annual Diurnal Temperature Range (DTR) and maximum Temperature (T_{max}) trend, observed in southern India and north-east region, are statistically significant. Likewise, the negative trend of DTR and minimum temperature (T_{min}) observed in north-west region is statistically significant. From these results, it can be inferred that the positive trend and negative trend of DTR is directly related to T_{max} and T_{min} respectively. Extremes of T_{max} like SU, TX10p, TX90p and WSDI shows statistically significant trend in southern India. Moreover, all the extremes except TX10p show significant trend in north-east India. Likewise the statistical significance of the extremes of T_{min} is also analysed and few grids shows statistical significance. However, a statistically non-significant trend cannot be judged as ‘no trend’. It only indicates that there is no evidence for that particular trend.

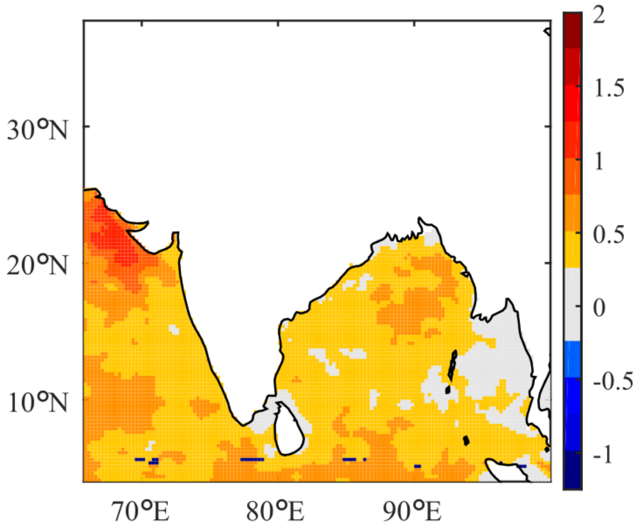

Figure S7: Ensemble Empirical Mode Decomposition trend for annual sea surface temperature (°C) for the period of 1982-2010. ±0.25 ˚C range is assigned a light grey color for easy distinction of positive and negative trends. The map was generated using the software MATLAB (version 2014b) <http://www.mathworks.com/products/matlab/>

Table S1: Climate Extreme Indices

Abbreviations	Definitions
SU (Summer days)	Number of days, when maximum temperature > 30°C
TX10p (Warm nights)	Number of days, when maximum temperature < 10th percentile
TX90p (Warm days)	Number of days, when maximum temperature > 90th percentile
WSDI (Warm spell duration index)	Total number of days with at least 6 consecutive days, when maximum temperature > 90th percentile
TR (Tropical nights)	Number of days, when minimum temperature > 20°C
TN10p (Cold nights)	Number of days, when minimum temperature < 10th percentile
TN90p (Cold days)	Number of days, when minimum temperature > 90th percentile
CSDI (cold spell duration index)	Total number of days with at least 6 consecutive days, when minimum temperature < 10th percentile
DTR (Diurnal Temperature Range)	Annual/Seasonal mean difference of maximum and minimum temperature

1 Methodology

The presence of trend, if any, in the variables, is deduced using Multi-dimensional Ensemble Empirical Mode Decomposition (MEEMD) method and the significance of these trends are evaluated by Monte Carlo Simulation. The MEEMD method is described below:

1.1 Multi-dimensional Empirical Mode Decomposition (MEEMD)

The presence of any trend in each of the variables is evaluated using MEEMD[1], which is developed from EEMD (Ensemble Empirical Mode Decomposition) and EMD (Empirical Mode Decomposition)[2]. EMD is a one dimensional, non-linear and non-stationary time domain decomposition method[3]. It is an adaptive and highly localized method, which decomposes a time series into multiple empirical modes known as intrinsic mode functions (IMFs). IMFs are simple oscillatory functions with certain amplitude and frequency, which is often related to a specific physical process. Moreover, IMFs have to satisfy two conditions:

i. The number of local extrema and the number of zero crossings must be equal or at most vary by one, while the function being symmetric in time.

ii. The mean value of the envelope defined by the local maxima and local minima must be equal to zero.

Generally, a time series is composed of two main components: mono component (*I*) and slow varying component (*R*). Mono component, also called as IMF, can be extracted through a refining process called sifting, through which the first IMF can be obtained. The steps involved in the sifting-process is shown in Figure S1. The entire process will stop when the slow varying component is a monotonic function or a curve contains at most one extremum at which no more oscillatory component can be defined. The original time series can be expressed as:

$$Y(t) = \sum_{j=1}^{n} I(t)_j + R(t)_n$$

As a rule, EMD is enforced through the sifting process, which entirely depends on the distribution of extrema. If there is any changes in the locations and values of extrema, it could lead to significantly different results. Moreover, oscillations of disparate scales may cause mode mixing[4,5], which leads to wrong interpretation of physical meaning of IMF. This clearly emphasizes that EMD is sensitive to noise. Since we know that real data mostly contain certain amount of random noise and intermittences, EMD approach is unstable and does not satisfy the physical uniqueness of the decomposition methods. To overcome this drawback, Ensemble Empirical Mode Decomposition (EEMD)[2,4,5] was developed, which is a noise assisted data analysis method. The steps involved in EEMD is described below:

I. Add a white noise series to the targeted data;
II. Decompose the data with added white noise into IMFs (as explained in EMD)
III. Repeat step I and step II several times with different white noise series added each time
IV. Obtain the (ensemble) mean of corresponding IMFs of the decompositions as the final result.

EEMD approach significantly reduces the chance of mode mixing and leads to the stable decomposition. Moreover, this method expresses the physical process at any given time, well-preserving the local behaviour[5–7]. Generally, input noise level is in the range of 0.1 to 0.4. We chose an amplitude of 0.2 times standard deviation of the corresponding data, as suggested by ref. 4. Moreover, the various number of ensembles are tried and finally ensemble size is chosen as 1000, which stabilizes the variability. This approach is further developed to handle both spatial and temporal locality known as MEEMD. Ref. 7 has analyzed three neighboring grids and observed that MEEMD satisfactorily replicates the difference of each original series and the respective components.

2 Seasonal EEMD Trend Variation of T_{max} and T_{min}

We assessed the evolution of T_{max} and T_{min} further for these four seasons i.e., Winter (DJF: December to February), Pre-monsoon (MAM: March to May), Summer Monsoon (JJA: June to August) and Post Monsoon (SON: September to November). Figure S2 shows the evolution of T_{max} for different seasons. During winter, an evident intensification (in terms of both area and magnitude) of positive trend from southern tip to the inner parts of the region is observed (Figure S2, top panel). Negative trend is seemingly confined to the foot-hills of Himalayas, with increased magnitude, however. Intensification of positive trend explains the reason for increase in DTR in most of the grids, an unexpected pattern during winters. Pre-monsoon season displays more dominant positive pattern to that of the winter, with remarkably high rate of increase in magnitude and spatial spread (Figure S2, 2nd panel). A brief relief is observed during monsoon season, with slight increase (decrease) in negative (positive) trend (Figure S2, 3rd panel). Nevertheless, the magnitude of positive trend increases by approximately 2˚C in the northeastern region by 2010. Increase in both spatial extent and magnitude of positive trend over north-east regions and Central India are detected during post-monsoon season also, though not much remarkable (Figure S2, bottom panel). Contribution from changes in T_{min} is investigated further as shown in Figure S3. Weakening of negative trend and intensification of positive trend are discernible during winter (Figure S3, top panel). Reversal of trends happened over north-west India and Himalayan foothills. Similar patterns are observed during pre-monsoon, monsoon and post-monsoon seasons (Figure S3, 2nd, 3rd and bottom panels respectively), with varying spatial extents and magnitudes.

3 Physical Interpretation

A brief analysis is performed to construe the causes for the diurnal asymmetry of surface temperature in India. Many studies[8–14] have attributed the change in trend of DTR to feedback processes like cloud cover, precipitation, soil moisture, sunshine duration and leaf

area index. All the above processes are inversely correlated with DTR except sunshine duration. However, these feedback processes are local in nature, which are consequently stimulated by some global factors. Recently ref. 15 attempted to simulate changes in DTR using two different models: natural and natural with anthropogenic forcings. They found that the later model could closely capture the diurnal asymmetry pattern. Since the variability of T_{min} in India is much influenced by anthropogenic gases and aerosol loading[16], the negative trend in DTR is correlated to the cloud covering formed due to aerosol loading in north India, especially in winter and pre-monsoon, when the aerosol loading is high. However, the same hypothesis does not seem to be appropriate for southern India, where DTR shows positive trend with increase in cloud cover[9,10,16]. Ref. 10 suspected that the sea surface temperature might be the reason behind this. Additionally, we observed that the trends are not biased either by urban or by altitude of the location and the warming is pronounced along the Coastal regions. Recent studies also have shown a significant correlation between the near surface air temperature and Sea Surface Temperature (SST)[17,18]. Therefore, we analyzed the trend of SST for the period 1982 to 2010 using Advanced Very High Resolution Radiometer (AVHRR) satellite SST gridded data of resolution 0.25˚× 0.25˚[19]. Usually, Bay of Bengal is warmer than Arabian Sea[20]. However, Figure S7 reveals that the rate of increase in positive trend of SST in Arabian Sea is higher than that of Bay of Bengal, which is also observed by a recent study[21]. Especially higher rate of positive trend up to 2˚C is observed in the northern-most part of west Coast. These places are surrounded by Pakistan and north-west India, which are arid regions and covered with dust clouds. The recent increase of SST resulted in the increase of moisture content in the atmosphere[22], which may be the possible reason for the recent increase in the minimum temperature, since the moisture in the atmosphere reduces the overnight cooling. Moreover, the southern west Coast shows increasing trend of maximum temperature than minimum temperature, which can also be attributed to the SST

and the strengthening of northern easterlies which transport the cool winds from north to south during winter[18]. Thus, it can be inferred that although the influence of a warmed up ocean is similar over the arid desert in the north-west and the equatorial forest along the west Coast, the trend in DTR is contradictory to each other because of the influence of its regional climate. However, a proper conclusion cannot be drawn from this analysis and needs simulation of climatic model driven by all the factors.

References

1. Wu, Z., Huang, N. E. & Chen, X. The Multi-Dimensional Ensemble Empirical Mode Decomposition Method. *Adv. Adapt. Data Anal.* **1,** 339–372 (2009).
2. Wang, Y. H., Yeh, C. H., Young, H. W. V, Hu, K. & Lo, M. T. On the computational complexity of the empirical mode decomposition algorithm. *Phys. a-Statistical Mech. Its Appl.* **400,** 159–167 (2014).
3. Huang, N. E. *et al.* The empirical mode decomposition and the Hilbert spectrum for nonlinear and non-stationary time series analysis. *Proc. R. Soc. A Math. Phys. Eng. Sci.* **454,** 903–995 (1998).
4. Wu, Z. & Huang, N. E. Ensemble Empirical Mode Decomposition: A noise-assisted data analysis method. *Adv. Adapt. Data Anal.* **1,** 1–41 (2009).
5. Wu, Z. & Huang, N. E. A study of the characteristics of white noise using the empirical mode decomposition method. *Proc. R. Soc. A Math. Phys. Eng. Sci.* **460,** 1597–1611 (2004).
6. Wu, Z., Huang, N. E., Wallace, J. M., Smoliak, B. V. & Chen, X. On the time-varying trend in global-mean surface temperature. *Clim. Dyn.* **37,** 759–773 (2011).
7. Ji, F., Wu, Z., Huang, J. & Chassignet, E. P. Evolution of land surface air temperature trend. *Nat. Clim. Chang.* **4,** 462–466 (2014).
8. Dai, A., Trenberth, K. E. & Karl, T. R. Effects of Clouds, Soil Moisture, Precipitation, and Water Vapor on Diurnal Temperature Range. *J. Clim.* **12,** 2451–2473 (1999).
9. Roy, S. Sen & Balling, R. C. Analysis of trends in maximum and minimum temperature, diurnal temperature range, and cloud cover over India. *Geophys. Res. Lett.* **32,** 1–4 (2005).
10. Jaswal, A. K. Recent winter warming over India - spatial and temporal characteristics of monthly maximum and minimum temperature trends for January to March. *Mausam* **61,**

163–174 (2010).

11. Rai, A., Joshi, M. K. & Pandey, A. C. Variations in diurnal temperature range over India: Under global warming scenario. *J. Geophys. Res. Atmos.* **117,** 1–12 (2012).

12. Peralta-Hernandez, A. R., Balling Jr, R. C. & Barba-Martinez, L. R. Analysis of near-surface diurnal temperature variations and trends in southern Mexico. *Int. J. Climatol.* **29,** 205–209 (2009).

13. Zhou, L. *et al.* Spatial dependence of diurnal temperature range trends on precipitation from 1950 to 2004. *Clim. Dyn.* **32,** 429–440 (2009).

14. Shen, X. *et al.* Spatiotemporal change of diurnal temperature range and its relationship with sunshine duration and precipitation in China. *J. Geophys. Res. Atmos.* **119,** 13,163-13,179 (2014).

15. Zhou, L., Dickinson, R. E., Dai, A. & Dirmeyer, P. Detection and attribution of anthropogenic forcing to diurnal temperature range changes from 1950 to 1999: comparing multi-model simulations with observations. *Clim. Dyn.* **35,** 1289–1307 (2010).

16. Kothawale, D. R., Kumar, K. K. & Srinivasan, G. Spatial asymmetry of temperature trends over India and possible role of aerosols. *Theor. Appl. Climatol.* **110,** 263–280 (2012).

17. Kothawale, D. R., Munot, A. A. & Krishna Kumar, K. Surface air temperature variability over India during 1901–2007, and its association with ENSO. *Clim. Res.* **42,** 89–104 (2010).

18. Revadekar, J. V., Varikoden, H., Murumkar, P. K. & Ahmed, S. A. On the relationship between sea surface temperatures, circulation parameters and temperatures over west coast of India. *Sci. Total Environ.* **551–552,** 175–185 (2016).

19. Reynolds, R. W. *et al.* Daily High-Resolution-Blended Analyses for Sea Surface

Temperature. *J. Clim.* **20,** 5473–5496 (2007).

20. Shenoi, S. S. C., D, S. & R, S. S. Why is Bay of Bengal warmer than Arabian Sea during the summer monsoon? *M* (2004). at <http://repository.ias.ac.in/75981/>

21. Kothawale, D. R., Munot, A. A. & Borgaonkar, H. P. Temperature variability over the Indian Ocean and its relationship with Indian summer monsoon rainfall. *Theor. Appl. Climatol.* **92,** 31–45 (2008).

22. Rana, A. S., Zaman, Q., Afzal, M. & Haroon, M. A. Characteristics of Sea Surface Temperature of the Arabian Sea Coast of Pakistan and Impact of Tropical Cyclones on SST. *Pakistan J. Meteorol.* **11,** 61–70 (2014).
